# The Impact of Uterus-Derived Prostaglandins on the Composition of Uterine Fluid During the Period of Conceptus Elongation in Dairy Heifers

**DOI:** 10.3390/ijms26051792

**Published:** 2025-02-20

**Authors:** Beibei Zhang, Yuan Han, Shengxiang Wang, Ming Cheng, Longgang Yan, Dong Zhou, Aihua Wang, Pengfei Lin, Yaping Jin

**Affiliations:** 1 Key Laboratory of Animal Biotechnology of the Ministry of Agriculture, College of Veterinary Medicine, Northwest A&F University, Yangling 712100, China; 2021060265@nwafu.edu.cn (B.Z.); 2021065023@nwafu.edu.cn (Y.H.); wsxwsx@nwafu.edu.cn (S.W.); 984737409@nwafu.edu.cn (M.C.); yanlonggang@nwafu.edu.cn (L.Y.); zhoudong1949@nwafu.edu.cn (D.Z.); wangaihua@nwafu.edu.cn (A.W.); 2 Department of Clinical Veterinary Medicine, College of Veterinary Medicine, Northwest A&F University, Yangling 712100, China; 3 Department of Preventive Veterinary Medicine, College of Veterinary Medicine, Northwest A&F University, Yangling 712100, China

**Keywords:** cattle, uterine fluid, meloxicam, prostaglandins, embryo elongation

## Abstract

In ruminants, the survival and development of the conceptus are heavily dependent on the composition of the uterine lumen fluid (ULF), which is influenced by prostaglandins (PGs). However, the variations in underlying PG-mediated ULF remain unclear. Herein, cycling heifers received an intrauterine infusion of vehicle as a control (CON) or meloxicam (MEL) on days 12–14 of the estrous cycle. Then, the ULF was collected on day 15 and alternations in its protein and lipid levels were analyzed. The suppression of prostaglandins induced by meloxicam resulted in 1343 differentially abundant proteins (DAPs) and 59 differentially altered lipids. These DAPs were primarily associated with vesicle-mediated transport, immune response, and actin filament organization, and were mainly concentrated on the ribosome, complement and coagulation cascades, cholesterol metabolism, chemokine signal pathway, regulation of actin cytoskeleton and starch and sucrose metabolism. These differential lipids reflected a physiological metabolic shift as the abundance of cell membrane-related lipids was modulated, including an accumulation of triacylglycerols and reductions in lysophosphatidylcholines, hexosyl ceramides, ceramides, and sphingomyelins species. Integration analysis of the DAPs and differentially altered lipid metabolites revealed that glycerophospholipid metabolism and choline metabolism were the core pathways. These findings highlight the potential roles of prostaglandins in ULF, providing new insights into the contributions of prostaglandins in the development of the conceptus.

## 1. Introduction

Early embryonic loss is a widespread issue in ruminants that significantly affects reproductive efficiency [1,2]. A significant proportion of pregnancy losses is due to the inability of the maternal uterus to support embryo growth and implantation [3,4,5]. After the insemination of a bovine (day 0), the spherical blastocysts hatch from the zona pellucida on days 9–10. The structures subsequently develop an ovoid or tubular form on days 12–14, and are then defined as the conceptus. The conceptus further develops into a filamentous shape and initiates the implantation process on days 16–17 [6,7,8]. Before implantation, the survival and elongation of the conceptus are highly dependent upon the ULF (termed histotroph); this process does not occur in vitro [9]. The loss of pregnancy in dairy cows mainly occurs in the first three weeks, especially during the period of conceptus elongation [8,10,11]. Therefore, understanding the formation and regulation of the ULF is important for improving fertility.

The substances in ULF are primarily derived from transport, synthesis and secretion by endometrial glandular epithelial (GE) and luminal epithelium (LE) cells [12]. The ULF is also metabolically semi-autonomous due to the activities of various enzymes [6,13]. The endometrium is collaboratively regulated by progesterone (P_4_) from the maternal system and by some signaling factors, secreted from the peri-implantation conceptus and endometrium. For instance, interferon tau (IFNT) and PGs support conceptus elongation and survival, as well as the regulation of endometrial receptivity formation [8,10,14,15]. PGs are lipid hormones that are synthesized by the conceptus and endometrium during early pregnancy in ruminants. In bovines, the concentrations of various PGs in the uterine fluid rapidly increase from days 12 to 18 of the pregnancy and the estrous cycle [16]. There are four principal bioactive prostaglandins in the bovine uterine lumen: prostaglandin E2 (PGE_2_), prostaglandin F2α (PGF_2α_), 6-keto-PGF1α (a stable metabolite of PGI_2_), and prostaglandin D2 (PGD_2_) [16,17]. An optimal ratio between PGE_2_ and PGF_2α_ in the uterine fluid is essential for establishing endometrial receptivity in ruminants, as PGE_2_ exhibits properties opposite to those of PGF_2α_ [18,19]. PGF_2α_ is the luteolytic hormone that induces both functional and structural luteolysis, whereas PGE_2_ is considered a luteoprotective mediator. The corpus luteum produces PGE_2_ in response to the endometrial PGE_2_, induced by IFNT or pregnancy, which serves as a luteoprotective mechanism that prolongs the corpus luteum’s lifespan [20,21,22]. In ruminants, PGF_2α_ is secreted and transported from the endometrium to the uterine vein during luteolysis, and from the endometrium to the uterine lumen, during maternal recognition and pregnancy establishment in ruminants [20].

Prostaglandin endoperoxide synthase 2 (PTGS2), a key cyclooxygenase and a rate-limiting enzyme in PG synthesis, is expressed in the endometrium and the trophectoderm of the elongated conceptus, and is induced by P_4_ and IFNT [17,23]. When the PGs were inhibited due to the intrauterine infusion of MEL—a selective inhibitor of PTGS2 (13.1 times more effective in inhibiting COX-2 compared with COX-1) [24,25]—conceptus elongation was inhibited in sheep [26]. In heifers, the pregnancy rate was dramatically reduced upon the receival of the MEL treatment 15 days after insemination [27]. PTGS2 expression is also considered a predictor for the successful bovine blastocyst development on day 7 [28]. Moreover, the synthesis of PGs is disrupted in the endometria of women with repeated in vitro fertilization failure [29]. Together, these studies indicate that PG signaling promotes conceptus elongation. However, there is limited information on the effect of the PGs on the composition of the uterine fluid. The abundance of PGs in the uterine fluid is significantly different between high-fertile and subfertile heifers [17]. The differential expression of prostaglandin-related genes and/or the abundance of PGs in the ULF are associated with dairy cow fertility [17,30]. PGs regulate the expression of genes associated with the elongation and implantation in the endometrial epithelium, prior to pregnancy recognition in sheep [10,26,31,32]. The presence of multiple PGs often has greater physiological significance than when only a single type is detected [16,17]. Therefore, a comprehensive study of the effects of PGs from different sources on the uterine environment may provide a new insight for improving fertility in dairy cows.

This study aims to explore the alternations of proteins and lipids in the ULF of dairy heifers during the window of conceptus elongation when suppressing PGs levels with intrauterine perfusion of MEL, and identify the possible metabolic pathways, proteins and metabolites induced by endometrium-derived PGs that affect conceptus elongation and implantation. We hypothesized that PGs likely exert autocrine and possibly intracrine effects on the endometrium, modifying the uterine environment to support conceptus development and implantation in ruminants.

## 2. Results

### 2.1. Plasma Concentrations of Progesterone and Prostaglandins

**Figure 1:**
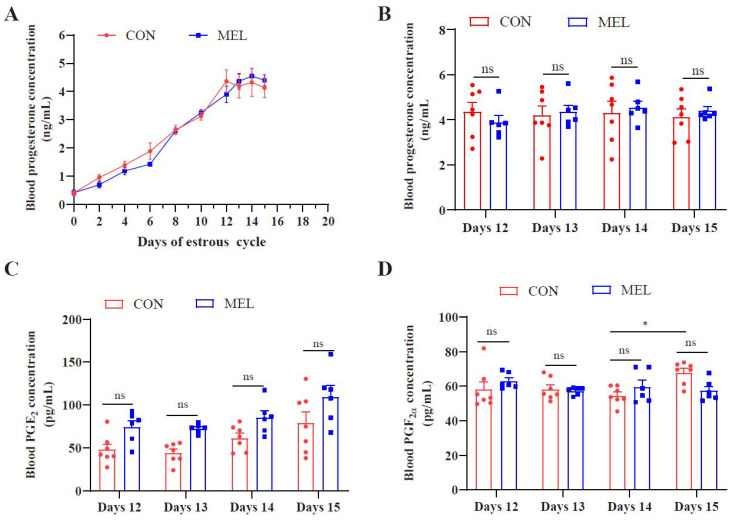
Hormone concentrations in blood samples. (**A**) Circulating concentrations of progesterone on different days, between the CON and MEL groups. (**B**) Plasma progesterone concentration between the CON and MEL groups. (**C**) Plasma PGF_2α_ concentration between the CON and MEL groups. (**D**) Plasma PGE_2_ concentration between the CON and MEL groups. “ns” as *p* > 0.05; “*” as *p* < 0.05.

Before perfusion, there was no significant difference in the circulating progesterone concentrations between the CON and MEL groups on the same day; however, levels increased following the formation of corpus luteum (CL) (Figure 1A). The PGE_2_, PGF_2α_ and P_4_ levels in blood showed no significant differences between the MEL and CON groups on days 12–15 post-treatment (Figure 1B–D). It is worth noting that the PGF_2α_ concentrations in the CON group significantly increased on day 15 compared with day 14.

### 2.2. MEL Treatment Altered the Protein Profile of the ULF in Dairy Heifers

**Figure 2:**
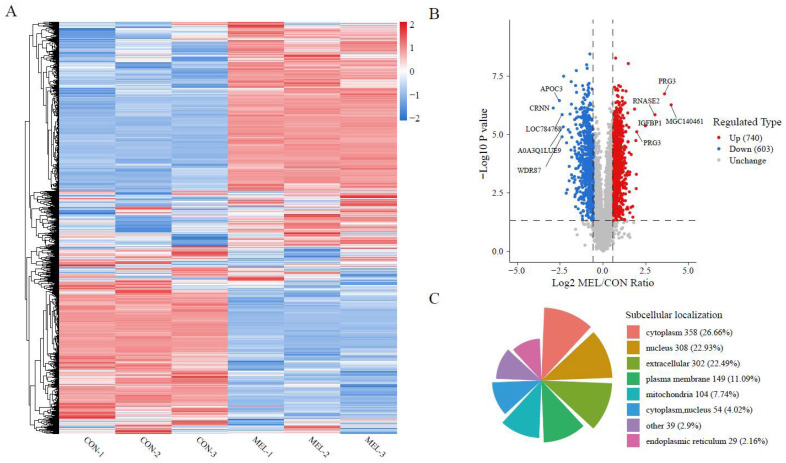
Proteomic profile of ULF samples using quantitative proteomics based on DIA technology. (**A**) The heatmap of all identified proteins between the CON and MEL groups. (**B**) Volcano plot of DAPs between the CON and MEL groups. (**C**) Subcellular localization map of DAPs between the CON and MEL groups.

To investigate the effects of the endometrium-derived PGs on the composition of uterine fluid, the protein profile of the ULF was analyzed after the intrauterine infusion of MEL. In total, 4055 and 4085 proteins were identified in the CON and MEL groups, respectively. The heatmap of the overall relatedness showed good biological replication within the groups and a striking separation between them (Figure 2A). Compared with the CON treatment, the MEL treatment generated 1343 DAPs (Table S1); 740 proteins were up-regulated and 603 proteins were down-regulated (Figure 2B), and these were mainly distributed in the cytoplasm (26.66%), nucleus (22.93%), extracellular (22.49%), plasma membrane (11.09%), and mitochondria (7.74%) (Figure 2C).

### 2.3. Biological Function Analysis of Differentially Abundant Proteins

**Figure 3:**
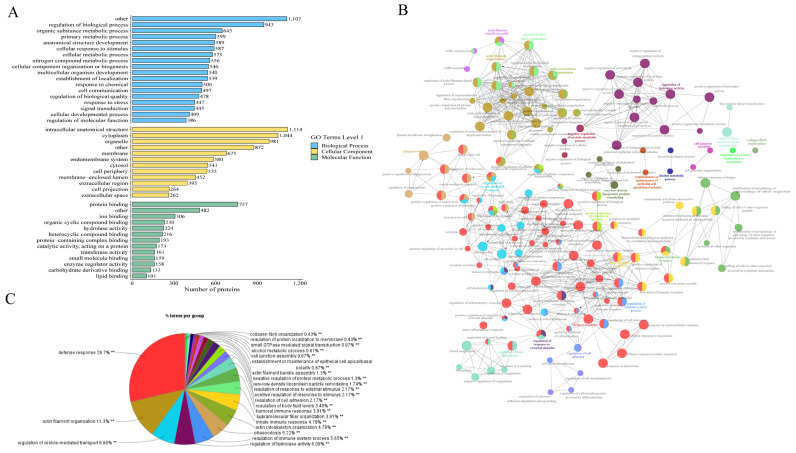
The analysis of DAPs between the CON and MEL groups. (**A**) GO enrichment analysis of DAPs between the CON and MEL groups. (**B**) The network diagram of biological processes of the DAPs between the CON and MEL groups. (**C**) The BP classification of the DAPs between the CON and MEL groups. “**” as *p* < 0.01.

GO analyses of the DAPs, shown in Figure 3A, revealed a significant enrichment in the biological processes, including the regulation of biological processes, primary metabolic processes, and the cellular response to a stimulus; enrichment in molecular function (protein binding, ion binding, hydrolase activity) and cellular components (intracellular anatomical structure, extra-cellular region) was also found. To gain insight into the biological function alterations induced after the MEL treatment, these DAPs were further analyzed using Cytoscape/ClueGo (v 3.9.1), based on the biological process results. The results showed that the DAPs were primarily related to the innate immune system, actin filament organization, regulation of vesicle-mediated transport, small GTPase-mediated signal transduction, proteolysis, very low-density lipoprotein particle remodeling, establishment or maintenance of epithelial cell apical/basal polarity, regulation of cell shape, regulated secretory pathway regulation and regulation of the response to an external stimulus (Figure 3B,C; Table S2). Among these terms, the regulation of vesicle-mediated transport and the response to external stimulus were the core biological processes that suggested that the PGs may play a crucial role in maternal–conceptus interactions and endometrial responsiveness to embryonic signals.

### 2.4. Pathway Enrichment Analysis of Differentially Abundant Proteins

**Figure 4:**
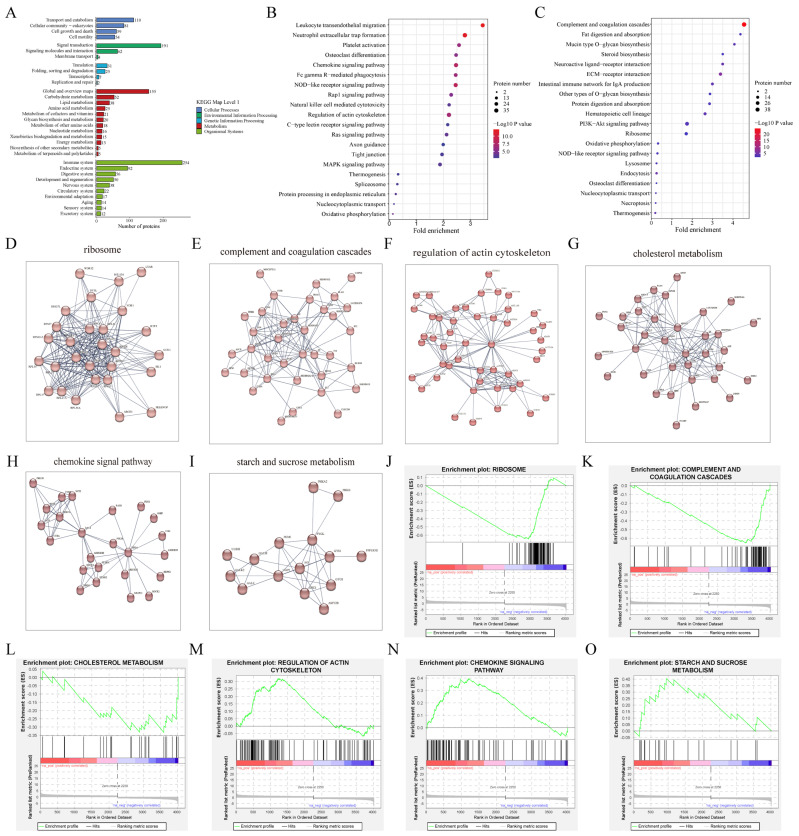
The enriched pathways of the DAPs between the CON and MEL groups. (**A**) The enriched KEGG pathways of the DAPs between the CON and MEL groups. (**B**) The top 20 KEGG pathways of the up-regulated DAPs in the ULF between the CON and MEL groups. (**C**) The top 20 KEGG pathways of the down-regulated DAPs in the ULF between the CON and MEL groups. The analyses of PPI with the DAPs involved in the (**D**) ribosome, (**E**) complement and coagulation cascades, (**F**) regulation of actin cytoskeleton, (**G**) cholesterol metabolism, (**H**) chemokine signal pathway, (**I**) starch and sucrose metabolism. The GSEA of the DAPs in the ULF between the CON and MEL groups, including the (**J**) ribosome, (**K**) complement and coagulation cascades, (**L**) cholesterol metabolism, (**M**) regulation of actin filament organization, (**N**) chemokine signal pathway, (**O**) starch and sucrose metabolism.

KEGG analysis showed that these DAPs were primarily enriched in the pathways related to the immune system, signal transduction, transport, and catabolism (Figure 4A; Table S3). Specifically, the up-regulated proteins were predominantly involved in the chemokine signaling pathway, the regulation of actin cytoskeleton, tight junction, Rap1 signaling pathway, and focal adhesion (Figure 4B), whereas the down-regulated proteins were enriched in complement and coagulation cascades, ECM–receptor interaction, the PI3K-AKT signaling pathway, protein digestion and absorption, and the ribosome (Figure 4C). To further analyze this protein profile, we used the STRING database to construct a protein–protein interactions (PPI) network, and identified the key functional modules via Markov cluster algorithm (MCL) analysis. These clusters primarily focused on the ribosome, complement, and coagulation cascades, chemokine signal pathway, regulation of actin cytoskeleton, cholesterol metabolism, and starch and sucrose metabolism (Figure 4D–I). The gene set enrichment analysis (GSEA) showed that the ribosome (normalized enrichment score (NES) = −4.77, *p*-value < 0.01) (Figure 4J), complement and coagulation cascades (NES = −4.62, *p*-value < 0.01) (Figure 4K), and cholesterol metabolism (NES = −1.68, *p*-value < 0.05) (Figure 4L) were significantly down-regulated after the MEL treatment, while the regulation of actin cytoskeleton (NES = 2.54, *p*-value < 0.05) (Figure 4M), chemokine signal pathway (NES = 2.93, *p*-value < 0.01) (Figure 4N), and starch and sucrose metabolism (NES = 1.79, *p*-value < 0.05) (Figure 4O) were significantly up-regulated.

### 2.5. MEL Treatment Altered the Overall Composition of Lipids in the ULF

**Figure 5:**
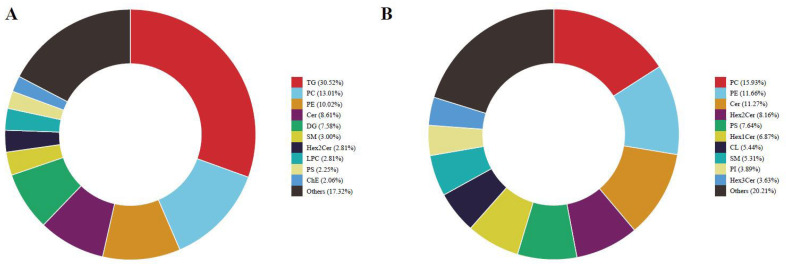
The relative abundance of the different lipid subclasses. Quantities of lipids class detected in the ULF between the CON and MEL groups: (**A**) the positive ion mode, (**B**) the negative ion mode. TG: triglyceride; PC: phosphatidylcholine; PE: phosphatidylethanolamine; Cer: Ceramides; DG: diglyceride; SM: sphingomyelin; Hex1Cer: hexosyl ceramide; Hex2Cer: Hex2-ceramide; Hex3Cer: Hex3-ceramide; LPC: lysophosphatidylcholine; PS: phosphatidylserine; ChE: cholesterol ester; CL: cardiolipin; PI: phosphatidylinositol.

The lipids in the ULF are crucial for the conceptus elongation in dairy cows [33]. Therefore, we further examined lipid metabolites changes in uterine fluid. A total of 1736 lipid molecules, categorized into 38 lipid classes, were identified in both positive and negative ion modes. Triglycerides (TGs) (*n* = 326) and phosphatidylcholines (PCs) (*n* = 262) were the most abundant lipids classes (Figure 5A,B; Table S4).

**Figure 6:**
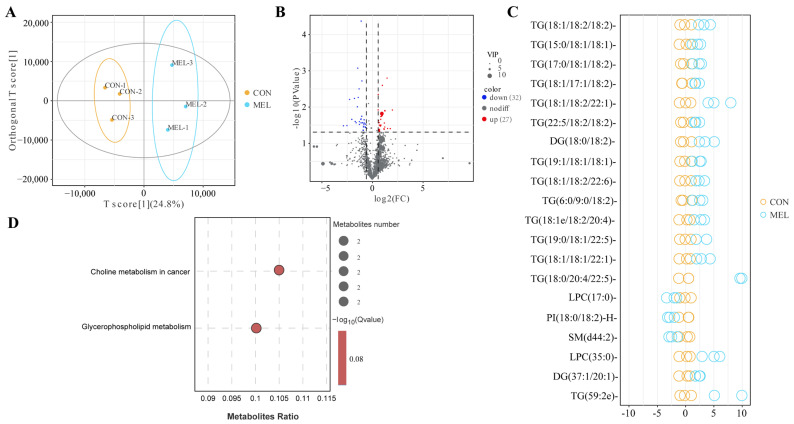
The analyses of the lipid profiles in the ULF between the CON and MEL groups. (**A**) The OPLS-DA analyses between the CON and MEL groups. (**B**) Volcano plot of different lipid metabolites between the CON and MEL groups. (**C**) The top 20 differential lipid metabolites based on the value of VIP. (**D**) Pathway enrichment analysis of differential lipid metabolites between the CON and MEL groups.

Orthogonal partial least squares discriminant analysis (OPLS-DA) revealed distinct sample distributions and differences between the CON and MEL groups (Figure 6A). Compared with the CON group, MEL treatment resulted in 59 differentially altered lipids (Figure 6B; Table S5), including 19 species of TG, 7 species of lysophosphatidylcholine (LPC), 6 species of dihexosyl N-acetylhexosyl ceramide (CerG3GNAc1), 6 species of hexosyl ceramide (Hex1Cer), 4 species of sphingomyelin (SM), 3 species of ceramides (Cer), 3 species of diglyceride (DG), 2 species of phosphatidylcholine (PC), 2 species of phosphatidylinositol (PI) 2 species of cardiolipin (CL), 1 species of monosialo trihexosyl ceramide (GM3), 1 species of phosphatidylserine (PS), 1 species of sphingosine bases (SPH), 1 species of phosphatidylglycerol (PG) and 1 species of lysophosphatidylethanolamine (LPE). It is noteworthy that the LPC, Hex1Cers and Cers classes were down-regulated and most of TGs were up-regulated in response to MEL. Among these differentially altered lipids, LPC (35:0), TG (19:0/18:1/22:5), TG (18:2/14:3/18:2), LPE (18:0e), TG (18:1/18:2/22:1), PC (18:3e/18:3), TG (59:2e), TG (6:0/9:0/18:2), CL (74:8)-H, and TG (18:0/20:4/22:5) exhibited a markedly greater abundance in the MEL group. In contrast, DG (21:5e), SM (d44:0), SM (d35:2), Cer (d18:1/23:0), Hex1Cer (d20:0/24:0), LPC (14:0), GM3 (d44:1+O), LPC (16:1e), PS (17:0/18:1)-H, and CerG3GNAc1 (d44:1) had lower concentrations in the MEL group (*p* < 0.05, Figure 6C). Moreover, the differential lipid metabolites were mainly enriched in glycerophospholipid metabolism and choline metabolism in cancer pathway (Figure 6D).

### 2.6. The Key Role of Prostaglandins in Influencing the Uterine Fluid

**Figure 7:**
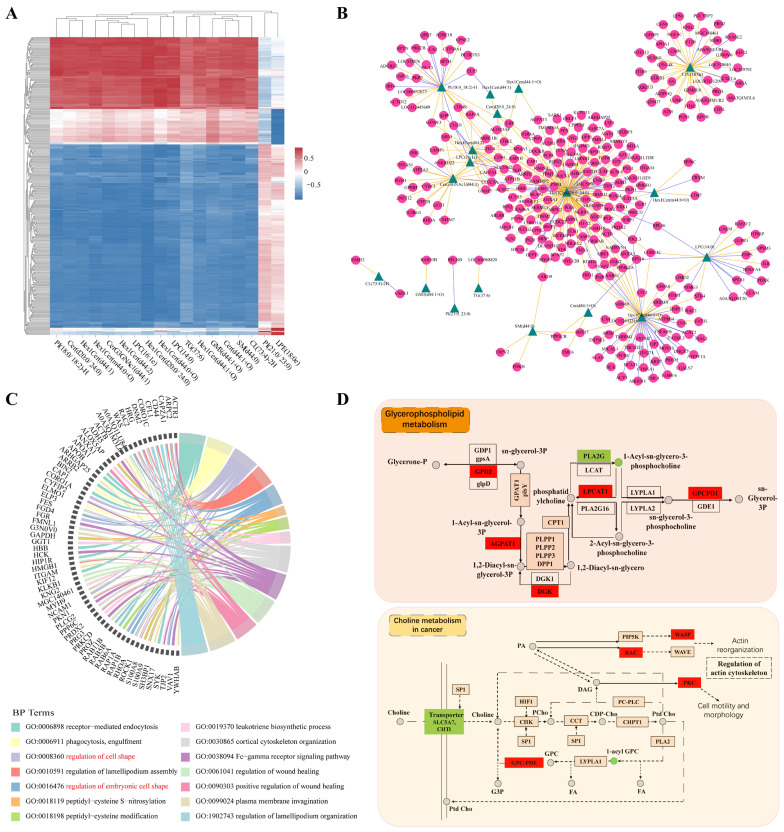
Integrative analysis of proteome and metabolome in the ULF between the CON and MEL groups. (**A**) The strong correlation heatmap of DAPs and the differential lipid metabolites between the CON and MEL groups (r ≥ 0.9 or r ≤ −0.9). (**B**) The correlation network of the DAPs and differential lipid metabolites between the CON and MEL groups. (**C**) Chord diagram of biological processes of the DAPs strongly related to differential lipid metabolites between the CON and MEL groups. (**D**) The common KEGG pathways between the DAPs and differential lipid metabolites.

The correlation between DAPs and different lipid metabolites may indicate functional or physical lipid–protein interactions. Therefore, we performed Spearman’s correlation analysis to examine the relationship between the DAPs and differential lipid metabolites. There were 357 protein–lipid pairs that showed very strong correlations (r ≥ 0.9 or r ≤ −0.9) (Figure 7A and Table S6). According to the network analysis, Hex1Cer (d20:0/24:0), Hex1Cer (d44:0+O), Hex1Cer (d44:2), LPE (18:0e), PI (18:0/18:2)-H, and CerG3GNAc1 (d44:1) interacted with the majority of the DAPs (Figure 7B). These proteins were mainly associated with cell migration (Rac family small GTPase 2 (RAC2), WASP actin nucleation promoting factor (WAS), coronin 1A (CORO1A), frabin (FGD4), myosin heavy chain 9 (MYH9), small GTPase RhoA (RHOA), Rho-associated coiled coil containing protein kinase 1 (ROCK1)), and the immune response (Vav guanine nucleotide exchange factor 1 (VAV1), spleen tyrosine kinase (SYK), protein kinase C Delta (PRKCD), phospholipase C gamma 2 (PLCG2), hematopoietic cell kinase (HCK), fetal growth restriction (FGR), cytoplasmic FMRP-interacting protein 2 (CYFIP2), beta-actin (ACTB), WAS, actin-related protein 2/3 complex subunit 2 (ARPC2), and actin-related protein 3 (ACTR3)) (Figure 7C). Glycerophospholipid metabolism and choline metabolism in cancer were identified as being commonly enriched pathways via the integrated proteome and lipid metabolite analysis (Figure 7D). Specifically, in terms of glycerophospholipid metabolism, the secretory phospholipase A2 (PLA2) exhibited a decreased accumulation in the MEL group, while the amount of lysophosphatidylcholine acyltransferase (LPCAT1) increased, leading to reduced levels of 1-acyl-sn-glycero-3-phosphocholine (1-acyl GPC).

## 3. Discussion

During the time of pregnancy recognition in ruminants, the IFNT secreted by the conceptus acts on the endometrium to inhibit the release of luteolytic pulses of PGF_2α_, thereby ensuring maintenance of the CL and the circulating P_4_ concentration [34]. Intrauterine infusion with meloxicam can inhibit uterine PGF_2α_ and PGE_2_ release in sheep due to COX-2 inhibition [35]. The P_4_ concentration in blood remained high in the MEL group, suggesting that intrauterine infusion with MEL could inhibit the secretion of PGs in the endometria of dairy cows. The blood concentration of PGE_2_ and PGF_2α_ remained almost unchanged in MEL group. However, the levels of prostaglandins and related metabolites, including 8-iso-13,14-dihydro-15-keto-PGF_2α_, prostaglandin F_1α_, 1a,1b-dihomo PGF_2α_, PGF_2α_ 1,15-lactone, 5-trans PGF_2α_, PGF_2α_ 1,11-lactone, 13,14-dihydro-15-keto-PGE2, 15-keto-PGE2, 19(r)-hydroxy-PGE1, ent-prostaglandin E2, PGI2, 6-keto-PGF1α, 15-deoxy-Δ12,14-PGD2, 8-iso-prostaglandin A2, prostaglandin B2 and TXB2, were significantly decreased after the MEL treatment. A previous study found that the effect of meloxicam on the concentration of 13,14-dihydro-15-keto-PGF (PGFM, a metabolic marker of PGF2α activity) in the serum was related to the body condition of the heifers [36]. Therefore, it is possible that higher doses of meloxicam can inhibit the circulating PGF_2α_ and PGE_2_.

In the study [17], the concentrations of PGE_2_, PGF_2α_ and 6-keto-PFG1α in the ULF were higher in highly fertile pregnant heifers than in subfertile heifers, but no differences were observed in the mRNA expression of selected PG synthases (PTGS2, PTGIS, PTGES, PTGFS) in the endometrium or conceptus. This is consistent with our findings, as although the abundances of PGs and their major metabolites in the uterine fluid were significantly reduced after the meloxicam treatment, the proteins related to prostaglandin synthesis were not found among our differentially abundant proteins (DAPs) (except phospholipase (PLA2G10)). However, we detected significant decreases in the abundances of proteins (PPARD, FABP3, PLTP) associated with the PPAR signaling pathway. PGI2 and PGJ2 are ligands for nuclear peroxisome proliferator-activated receptors (PPARs) [37]. PPARs are essential for conceptus development in ruminants [38,39], where they mediate lipid signaling in the conceptus by dimerizing with retinoid X receptors (RXRs) to regulate the transcription of target genes upon binding to PPAR-responsive elements (PPREs) [40]. Notably, the expression of fatty acid-binding protein 3 (FABP3) is elevated in pregnant heifers, compared to non-pregnant heifers, and in highly fertile pregnant heifers, compared to subfertile pregnant heifers [8,17]. FABP3 coordinates lipid responses, binds various lipids, including eicosanoids and long-chain fatty acids, and has been shown to regulate cell growth and proliferation [41]. FABP3 is up-regulated in the endometrial luminal epithelium on days 15 and 18 of pregnancy in cattle [42], indicating that FABP3 may play a key role in the influence of PGs on the uterine environment.

In this study, the MEL treatment-related ULF DAPs were primarily associated with vesicle-mediated transport, including that of nitrogen, acyl-CoA, aromatic compounds, fatty acids, carboxylic acid, and heterocyclic compounds, as well as proteins, amides, organic substances and bicarbonates. The ULF primarily originates from transport, synthesis, and secretion by the endometrial LE and GE [12,43]. The extracellular vesicles (EVs) in the ULF are essential mediators of maternal–conceptus communication and can serve as vehicles for the transport of selected compounds to support embryonic development, including proteins, lipids, enzymes, nucleic acids and metabolites [44,45]. In sheep, the number of EVs in the ULF increases during the onset of conceptus elongation. These alterations in EVs in the uterine lumen during the early pregnancy provide a mechanism to synchronize the embryo development with the dynamic remodeling of uterine endometrium [46]. Exosomes in the ULF enhance the developmental competence and quality of bovine embryos, produced through somatic cell nuclear transfer and in vitro fertilization embryos [47,48]. Therefore, these results imply that PGs are involved in the nutrient and ion mobilization and vesicle-mediated transport from the endometrium, which may be an important mechanism through which PGs influence conceptus development.

In addition to the secretion and transport from the endometrium, some of the metabolites in the ULF are formed via enzyme-mediated semi-autonomous metabolism [13]. Herein, the DAPs were significantly enriched in lipid and carbohydrate metabolism after the MEL treatment, including cholesterol metabolism and starch and sucrose metabolism. Lipids are important and abundant components in the ULF; the rapid proliferation of the trophectoderm requires large amounts of lipids for the synthesis of cell membranes, energy and cell signaling during the onset of elongation in ruminant [33,49]. Our targeted lipidomic analysis found that the inhibition of PGs leaded to the accumulation of TGs and decreased in the level of LPCs, Hex1Cers, Cers and SMs species in the ULF. LPCs, Cers and SMs are major components of the cell membrane. Under physiological conditions, the bovine ULF undergoes a metabolic shift during the onset of conceptus elongation that is primarily induced by P4. This shift includes increases in the levels of lipids associated with cell membrane, such as phospholipids, phosphatidylethanolamine and lysophospholipids [6]. This metabolic shift is consistent with a rapid accumulation of PGs in the ULF [16]. In this study, our findings further demonstrated that endometrium-derived PGs were critical for regulating the physiological metabolic shift in the ULF during the conceptus elongation period.

Correlation analysis of the DAPs and differential lipid metabolites revealed that the glycerophospholipid metabolism was a key metabolic pathway influenced by MEL treatment. Compared with subfertile heifers, most lipid metabolites were elevated in the ULF of pregnancy high-fertile heifers. This phenomenon was primarily linked to glycerophospholipids metabolism and steroid biosynthesis [50], which indicated that glycerophospholipid metabolism may be involved in dairy cow fertility. Glycerophospholipids are important components of cell membranes and also serve as precursors of eicosanoids, lysophospholipids and endocannabinoids. Saturated LPC, a major component of the cell membrane, is produced through the cleavage of phosphatidylcholine (PC) by phospholipase A2 (PLA2) or the transfer of fatty acids to free cholesterol by lecithin–cholesterol acyltransferase (LCAT) [51]. Additionally, LPC also can be converted back to PC via lysophosphatidylcholine acyltransferase (LPCAT) in the presence of acyl-CoA [52]. In this study, the abundance of PLA2 decreased, while that of LPCAT1 increased in the MEL group, which illustrated that the reduction in the levels of LPCs was partly due to the influence of PGs on the enzymic activity in the ULF. Furthermore, LPC (16:1e) very strongly correlated with these proteins related to cell migration and proliferation (GNAI3, CLEC5A, SLC35B2). LPCs are also a type of lysophospholipids and can be converted to lysophosphatidic acid (LPA) via a secretory enzyme [53]. In cows, LPAs play a crucial role in early embryo–maternal interactions, promoting embryonic survival [54]. Together, our results suggest that endometrium-derived PGs may influence conceptus development via regulating the abundance of LPCs in the ULF. Sphingolipids and Cers are also the important components of cell membranes and participates in cell proliferation, differentiation and apoptosis. Cers levels were significantly up-regulated in the ULF in of sheep on day 14 of pregnancy [55]. Therefore, the down-regulation of these compounds may affect embryo development. These findings suggest that PGs act as upstream regulators and are involved in the transport and metabolism of selected components in the ULF that are strongly associated with embryo development, during conceptus elongation.

The DAPs we identified were mainly associated with the immune system. The complement and coagulation cascades and chemokine signal pathway showed significant dysregulation after the MEL treatment. Mild immunosuppression and inflammation are essential for the maternal-conceptus communication [56]. Previous studies have demonstrated that physiological complement activation occurs during normal pregnancy in humans, bovines, pigs, and mice, and acts as a regulatory mechanism. Additionally, the complement cascade and coagulation cascade are closely interconnected, with each capable of activating the other reciprocally [57,58,59]. These results indicated that PGs were involved in maintaining the immune balance of the uterine microenvironment, mainly via the regulation of the complement and coagulation cascades and the chemokine signal pathway. Meanwhile, in over-conditioned dairy cows, the complement and coagulation cascades, as well as the acute inflammatory response, undergo significant changes during the transition period, compared with those in normal cows [60]. Therefore, combined with the above mentioned effects of PGs on lipid metabolism, these findings indicated that lipid metabolism and the immune response in the uterine environment may have ripple effects, and that PGs play a key role in maintaining immune and metabolic homeostasis during early pregnancy.

Moreover, the regulation of actin filament organization and the ribosome important factors for successful bovine pregnancies were also significantly enriched in response to the MEL treatment. Integrins bind extracellular matrix (ECM) molecules to mediate adhesion, transduce cell signals, and reorganize the cytoskeleton to stabilize adhesion. These functions are crucial for embryo implantation and placentation in all mammals [61]. A pervious study found that pregnancy loss in subfertile heifers is linked to the process of ECM remodeling, and that an excessive ECM could hinder embryonic adhesion to the endometrium [8]. In this study, the ECM–receptor interaction pathway was down-regulated after the MEL treatment, suggesting that regulation of ECM–receptor interactions may play a crucial role in the function of PGs as a necessary signal for successful implantation in dairy cows. Additionally, the ribosome serves as a major metabolic hub influencing cellular homeostasis and the development of multicellular organisms. Tissue development relies on ribosome homeostasis to support the determination and transition of cellular fate [62]. Therefore, the results further indicate that PGs can mediate dynamic endometrium remodeling, which is essential for endometrial receptivity formation.

The intricate composition of uterine fluid makes it highly suitable for an analysis using proteomics and metabolomics, enabling the identification of candidate genes involved in regulating conceptus elongation and implantation in ruminants. However, extracting biologically meaningful conclusions from large datasets remains a significant challenge in omics research. Therefore, future studies should integrate approaches to elucidate the mechanistic roles of specific factors governing conceptus elongation and uterine receptivity.

## 4. Materials and Methods

### 4.1. Animals and Experimental Design

All experiments in this study were carried out in accordance with the Guide for the Care and Use of Agricultural Animals in Agricultural Research and Teaching, and were approved by the Ethics Committee on the Use and Care of Animals at Northwest A&F University (No. 2021100903).

Dairy heifers (13 ± 1 months; BW (body weight): 360 ± 30 kg) were housed in a free field, and fed a young cow total mixed ration (TMR) once daily. All heifers were subjected to an estrous cycle synchronization program as in a previous report [63]. Briefly, the synchronization program was initiated by injection of PGF_2α_ on day −18; GnRH was injected on days −15, −8 and 0, respectively. Then, PGF_2α_ was injected on days −3 and −2, respectively. The day of the final injection of gonadotropin-releasing hormone (GnRH) was defined as day 0 of the estrous cycle. On this day, heifers were inseminated with sperm-free seminal plasma, which was obtained by removing sperm through centrifugation at 4000 rpm. Synchronous estrus was confirmed via transrectal ultrasonography assessment of the ovaries. When the ovaries presented a dominant follicle and did not contain corpus luteum (CL) on day 0, and there was an appearance of a CL on the same ovary of the dominant follicle on day 7, the cows were used in the subsequent experiments.

The experimental design is depicted in the Figure S1. In total, thirteen dairy heifers with successful synchronization of the estrous cycle were randomly assigned into two groups. The MEL group contained six dairy heifers that were intrauterinely infused with meloxicam solution (3 mg meloxicam (Sigma, St. Louis, MO, USA) that was initially dissolved in 300 μL dimethyl sulfoxide (DMSO) at a concentration of 10 mg/kg and then diluted with 5 mL phosphate-buffer saline (PBS)). The procedure was conducted via transcervical catheterization using an embryo transfer gun connected to a 10 mL syringe. Heifers in the CON group received intrauterine infusion of thevehicle (300 μL DMSO + 5 mL PBS). The intrauterine perfusion was administrated daily from day 12 for 3 days and uterine horn fluid was collected on day 15.

### 4.2. The Collection of Uterine Horn Fluid and Blood

On day 15, caudal epidural anesthesia was performed via the injection of 2% lidocaine hydrogen chloride (HCl) solution (Sichuan Jixing Animal Pharmaceutical Co., Ltd., Zigong, China) into the first coccygeal intervertebral space. The uterine fluid was collected in the uterine horn ipsilateral of the CL according to the method of the previous report with some modifications [63]. The uterine horn was flushed via transcervical catheterization using an embryo transfer gun connected to 50 mL syringe containing 30 mL PBS. Uterine lumen fluid was then recovered into a 100 mL syringe and immediately transferred to sterile tube. Samples with a recovered volume exceeding 15 mL and free from visible blood contamination were centrifuged at 4 °C and 2000× *g* for 20 min. The resulting supernatants were aliquoted into equal parts and immediately stored in liquid nitrogen.

Blood samples were collected from puncture of the coccygeal blood vessels using vacutainer collection tubes containing dipotassium ethylene diamine tetraacetic acid (EDTA). On days 12, 13 and 14, the blood samples were collected after intrauterine perfusion for 1 h. On day 15, the blood samples were collected as well as the uterine fluid. The blood samples were immediately placed on ice and subsequently centrifuged at 3000× rpm for 10 min at 4 °C to isolate plasma. All samples were then divided equally and stored at −80 °C.

### 4.3. The Detection of Progesterone and Prostaglandins

The concentration of progesterone in blood plasma samples was measured via Enzyme-Linked Immunosorbent Assay (ELISA) (002401, Jiangsu MEIMIAN Inc., Yancheng, China) according to the manufacturer’s protocol. The standard curve ranged from 0.1 to 100 ng/mL, with a sensitivity of 0.15 ng/mL. The intra- and inter-assay coefficients of variation were 8.7 and 11%, respectively. The concentration of PGE_2_ in blood plasma was measured using commercially available high-sensitivity competitive ELISA kits (514010, Cayman Chemical Company, Ann Arbor, MI, USA). The PGE2 standard curve ranged from 7.8 to 1000 pg/mL, and the median effective dose (ED 50) of the assay was 40–50 pg/mL. The intra- and inter-assay coefficients of variation (CVs) were 10 and 12%, respectively. The determination of PGF_2α_ concentration was performed using an ELISA kit (516011, Cayman Chemical Company, Ann Arbor, MI, USA). The standard curve ranged from 9.5 to 2000 pg/mL, the ED 50 of the assay was 9 pg/mL and the intra- and inter-assay CVs were, on average, 9.4% and 12%, respectively.

### 4.4. LC-MS/MS

The peptides were dissolved in a mobile phase A and separated using an EASY-nLC 1200 ultra-high performance liquid chromatography (UHPLC) system (#LC140, Thermo Fisher, Waltham, MA, USA). Mobile phase A consisted of water containing 0.1% formic acid and 2% acetonitrile; mobile phase B was water solution containing 0.1% formic acid and 90% acetonitrile. The liquid phase gradient was set as follows: 0–22.5 min, 6–22% B; 22.5–26.5 min, 22–34% B; 26.5–28.5 min, 34–80% B; 28.5–30 min, 80% B; The flow rate was maintained at 700 nL/min. The peptides were separated via ultra-performance liquid chromatography, ionized and analyzed using an Orbitrap Exploris 480. The ion source voltage was set at 2.3 kV. The Orbitrap detected both secondary fragments and peptide parent ions. The first mass spectrometry scanning range was set to 350–1400 *m*/*z* with the resolution of 60,000; while the secondary mass spectrometry scanning range started at 120 *m*/*z* with a resolution ratio of 15,000. Data acquisition was performed in data independent acquisition (DIA) mode.

### 4.5. Data Processing and Analyses

The resulting Tandem Mass Spectrometry (MS/MS) data were processed using the MaxQuant search engine (v.1.6.15.0). Tandem mass spectra were searched against the UniProt Bos taurus database (20230220) (37,508 sequences) concatenated with a reverse decoy database. Trypsin/P was specified as cleavage enzyme, allowing up one missed cleavage site. N-terminal me-thionine excision and cysteine-carbamidomethylation were set as fixed modifications. The false discovery rate (FDR) was controlled at <1% at both the peptide spectrum match (PSM) level and the protein level. Identified proteins were required to contain at least one unique peptide. All other MaxQuant parameters were set to their default values. DAPs were defined using fold change (FC) ≥ 1.5 or ≤1/1.5 with *p*-value < 0.05. The Gene Ontology (GO) and Kyoto Encyclopedia of Genes and Genomes (KEGG) pathway analyses were performed with DAPs, and pathway enrichment was conducted using GSEA according to a previous report [64]. The STRING database (version 11) was used to construct a protein–protein interaction network (PPI) of the DAPs, with visualization and analysis performed using Cytoscape 3.9.1. The biological processes of the DAPs were analyzed using the ClueGO plugin in Cytoscape [65]. Cluster analysis of DAPs was conducted using the Markov cluster algorithm with an inflation parameter value of 2.0 [66]. The hub proteins were identified using the cytoHubba plugin in the Cytoscape, based on their maximal clique centrality (MCC) scores [67].

### 4.6. Lipid Extraction and Targeted Lipidomics

Lipids were extracted using the Methyl-tert-Butyl Ether (MTBE) method as described previously [36]. Analyses were performed on an UHPLC Nexera LC-30A ultra-performance liquid chromatography system (SHIMADZU, Kyoto, Japan) coupled with Q-Exactive Plus (Thermo Scientific, Waltham, MA, USA). Solvent A consisted of acetonitrile–water (*v*/*v*, 6:4) containing 0.1% formic acid and 0.1 mM ammonium formate, and solvent B was acetonitrile–isopropanol (*v*/*v*, 1:9) containing 0.1% formic acid and 0.1 mM ammonium formate. The initial mobile phase was set to 30% solvent B with a flow rate of 300 μL/min and held for 2 min. The gradient then linearly increased to 100% solvent B in 23 min, followed by re-equilibrating in 5% solvent B for 10 min. Mass spectra were acquired using Q-Exactive Plus in positive and negative ion modes. ESI (Electron Spray Ionization) parameters were optimized and preset as follows: source temperature at 300 °C, capillary temperature at 350 °C, the ion spray voltage at 3000 V, S-Lens RF Level at 50% and a scan range of 200–1800 *m*/*z*. Lipid molecules and fragments were analyzed according to the following methodology: after each full scan, 10 fragments (MS2scan, HCD) were collected. The resolution of MS1 was set to 70,000 at *m*/*z* 200, and for MS2 it was set to 17,500 at *m*/*z* 200. LipidSearch software version 4.2 (Thermo Fisher Scientific, USA) was used for peaks extraction, the identification of lipid molecules and internal standards. The main parameters included a precursor tolerance of 5 ppm, a product tolerance of 5 ppm, and a product ion threshold of 5%.

### 4.7. Data Processing and Statistical Analyses

Lipid species were identified using the LipidSearch software (version 4.2, Thermo Scientific TM, Waltham, MA, USA) to process the raw data, including the peak alignment, retention time correction, and extraction of the peak area. For positive ion mode searches, adducts of +H and +NH4 were selected; while for negative mode searches, adducts of -H and +CH3COO were used, as ammonium acetate was included in the mobile phases. For the data extracted from LipidSearch, ion peaks with a value of >50%, missing from the group, were removed. Normalization and integration were performed using the Perato scaling method. Differentially altered lipids were identified as those with “FC ≥ 1.5 or ≤1/1.5 with *t*-test *p*-value < 0.05”. The GO and KEGG pathway analyses were conducted with differential lipid metabolites.

### 4.8. Statistical Analysis

The results are presented as the means ± SEMs obtained from at least three independent experiments. Statistical analyses were performed with GraphPad Prism software (version 8.0) using unpaired Student’s *t*-tests for experiments with two groups or one-way analysis of variance (ANOVA) for experiments with multiple groups. Statistically significant differences are indicated as follows: *p* < 0.05 (*), *p* < 0.01 (**), and *p* > 0.05 (no significance, ns).

## 5. Conclusions

In summary, the results of this study suggest that the composition of proteins and lipids in uterine lumen fluid is influenced by the abundance of PGs. The DAPs identified were mainly related to the vesicle-mediated transport, immune response, and endometrial remodeling. Meanwhile, a decrease in uterus-derived PGs suppressed the physiological metabolic shift via glycerophospholipid metabolism-mediated regulation of cell membrane lipid abundance. Overall, these findings indicate that uterus-derived PGs are important regulators of the uterine environment and provide a comprehensive understanding of the mechanisms through which the PGs from the endometrium can influence conceptus survival and development.

## Data Availability

Data are contained within the article and Supplementary Materials.

## Supplementary Materials

The following supporting information can be downloaded at: https://www.mdpi.com/article/10.3390/ijms26051792/s1.
