# Supplementary figures and images for: The Impact of Uterus-Derived Prostaglandins on the Composition of Uterine Fluid During the Period of Conceptus Elongation in Dairy Heifers
Source: Int J Mol Sci. 2025 Feb 20;26(5):1792. doi: 10.3390/ijms26051792 (PMC11899274; doi:10.3390/ijms26051792)

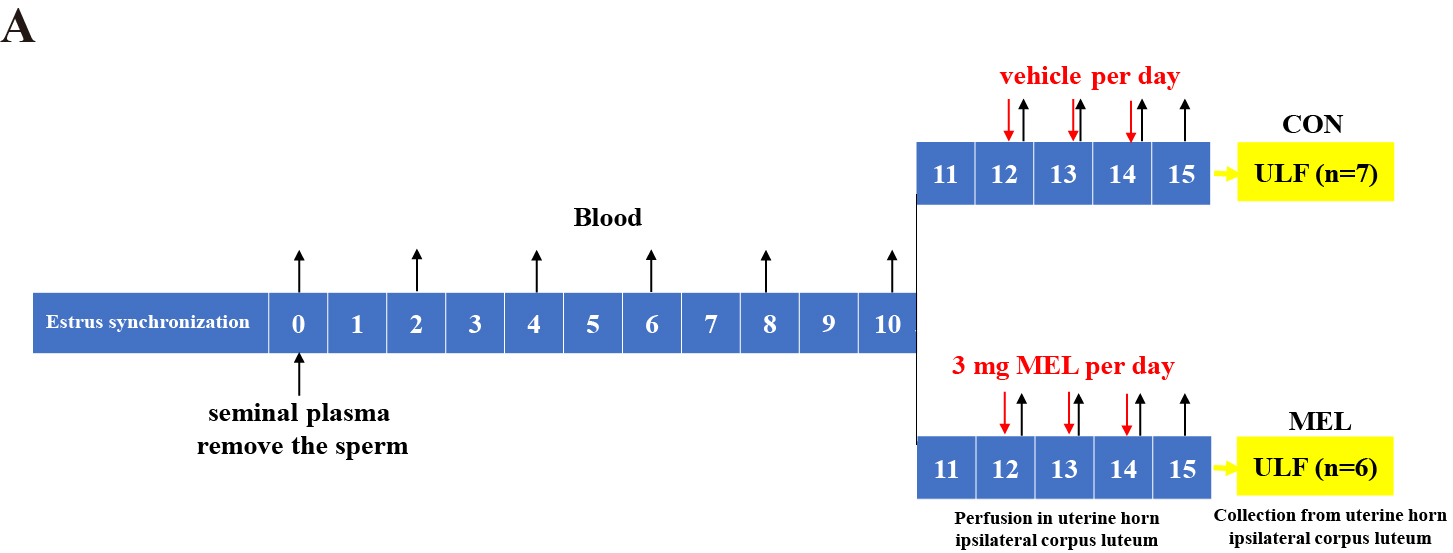

Supplement: Supplementary file 1 [file ijms-26-01792-s001.zip › Figure S1.jpg]
